# PKBγ/AKT3 loss-of-function causes learning and memory deficits and deregulation of AKT/mTORC2 signaling: Relevance for schizophrenia

**DOI:** 10.1371/journal.pone.0175993

**Published:** 2017-05-03

**Authors:** Kristy R. Howell, Kirsten Floyd, Amanda J. Law

**Affiliations:** 1 Department of Psychiatry, School of Medicine, University of Colorado, Aurora, Colorado, United States of America; 2 Department of Cell and Developmental Biology, School of Medicine, University of Colorado, Aurora, Colorado, United States of America; Stanford University School of Medicine, UNITED STATES

## Abstract

Psychiatric genetic studies have identified genome-wide significant loci for schizophrenia. The AKT3/1q44 locus is a principal risk region and gene-network analyses identify AKT3 polymorphisms as a constituent of several neurobiological pathways relevant to psychiatric risk; the neurobiological mechanisms remain unknown. AKT3 shows prenatal enrichment during human neocortical development and recurrent copy number variations involving the 1q43-44 locus are associated with cortical malformations and intellectual disability, implicating an essential role in early brain development. Here, we investigated the role of AKT3 as it relates to aspects of learning and memory and behavioral function, relevant to schizophrenia and cognitive disability, utilizing a novel murine model of Akt3 genetic deficiency. Akt3 heterozygous (Akt3^-/+^) or null mice (Akt3^-/-^) were assessed in a comprehensive test battery. Brain biochemical studies were conducted to assess the impact of Akt3 deficiency on cortical Akt/mTOR signaling. Akt3^-/+^ and Akt3^-/-^ mice exhibited selective deficits of temporal order discrimination and spatial memory, tasks critically dependent on intact prefrontal-hippocampal circuitry, but showed normal prepulse inhibition, fear conditioned learning, memory for novel objects and social function. Akt3 loss-of-function, reduced brain size and dramatically impaired cortical Akt Ser^473^ activation in an allele-dose dependent manner. Such changes were observed in the absence of altered Akt1 or Akt2 protein expression. Concomitant reduction of the mTORC2 complex proteins, Rictor and Sin1 identifies a potential mechanism. Our findings provide novel insight into the neurodevelopmental role of Akt3, identify a non-redundant role for Akt3 in the development of prefrontal cortical-mediated cognitive function and show that Akt3 is potentially the dominant regulator of AKT/mTOR signaling in brain.

## Introduction

Schizophrenia is a common neuropsychiatric disorder, characterized by positive (i.e. hallucinations, delusions) and negative symptomatology (i.e. flat affect, social withdrawal, lack of motivation) and cognitive disability. Deficits ascribed to abnormal development of the prefrontal cortex (PFC) and hippocampal formation, including working memory, executive function and cognitive flexibility, represent core features of the illness with an unknown etiology [1–4].

Large-scale genetic studies of schizophrenia have identified several genomic loci and gene pathways that increase risk. A recent genome-wide association study (GWAS) of 36,989 cases and 113,075 controls identified 108 independent associations [5], and network analyses of these data identified risk in several gene pathways involved in neuronal, immune and histone biology [6]. Interestingly, overlap of risk loci and antipsychotic drug gene targets has recently been reported [7], suggesting a link between disease etiology and antipsychotic mechanisms of action. Given the increasing understanding of the genetic basis of schizophrenia, a critical next step in mental illness research is identification of mechanisms and characterization of the *in vivo* function of risk genes. Such validation is necessary to identify novel therapeutic targets of pathophysiological relevance.

Genetic variation in the AKT3 locus (chr1:243503719–244002945) is a top GWAS signal in schizophrenia [5,8] and pathway analysis identified 50 single nucleotide polymorphisms (SNPs) within the AKT3 gene that contribute to four of the top pathways associated with risk for schizophrenia and bipolar disorder [6]. Moreover, recent investigations testing for enrichment of the 108 schizophrenia-risk loci, and overlapping rare singleton disruptive mutations in gene sets coding for proteins targeted by antipsychotic drugs, identified AKT3 as a potential target gene of relevance to antipsychotic treatment and response [7]. These studies identify AKT3 as a promising risk gene for schizophrenia and further highlight the AKT signaling pathway as a potential target for improved treatment development [9–11].

AKT (also known as protein kinase B (PKB)) is a critical intracellular serine/threonine kinase that translates signals from extracellular stimuli including growth factors, cytokines and neurotransmitters in response to activation of the intracellular second messenger, phosphatidylinositol 3-kinase (PI3K). AKT signaling plays critical roles in organogenesis, influencing cell growth, proliferation, survival, differentiation and metabolism [12–14] and in mammals, consists of a tripartite pathway which includes members AKT1, AKT2, and AKT3, each with a high level of protein homology [15] and encoded by independent genes (14q32.32; 19q13.2 and 1q44, respectively). The key regulatory phosphorylation sites on AKT (Ser^473^and Thr^308^) are conserved across isoforms and recent evidence suggests that phosphorylation of Ser^473^, which is governed by mTORC2, determines signaling to specific substrates [16,17]. Although the cellular function of each isotype is poorly understood, emerging in-vitro data suggests that AKT isoforms are present at distinct subcellular locations [18,19], exhibit different substrate specificity and importantly enzyme activity, with AKT3 being 15–40 fold more active than AKT1 or AKT2 [20,21].

Murine studies of single and double isoform Akt knockout (KO) mice have also provided critical information on the developmental role of individual Akt isoforms, with Akt1 being a key regulator of whole body organismal growth [22]; Akt2 being critical for normal glucose homeostasis [23], and Akt3 playing a specific role in attainment of normal brain size, specifically through regulation of cell size and number [24,25]. Consistently, recent data in rat stroke models demonstrate a critical neuroprotective role for Akt3 [26].

In addition to the recent AKT3 association, genetic variation in AKT1 has previously been implicated in risk for neuropsychiatric phenotypes. Specifically, AKT1 is associated in humans with schizophrenia [27–33] and abnormal PFC and hippocampal- structure and function [34–37]. Furthermore, evidence from postmortem brain and peripheral cells of patients with schizophrenia has identified reduced AKT activity, either through reduced expression of AKT1 [27,30], AKT3 [38] or phosphorylated AKT Ser^473^ [39,40]. It is important to note that it is currently unknown whether the decrease in AKT Ser^473^ phosphorylation observed in patients with schizophrenia [39,40] is accounted for by alterations in the activity of AKT1, AKT2 or AKT3.

In this context, mice with genetic deletion of Akt1 or Akt2 have previously been examined for neurobehavioral deficits relevant to psychiatric illness. Akt1 deficiency results in deficits of sensorimotor gating, hippocampal/PFC-dependent learning (affecting contextual fear and spatial and working memory), synaptic plasticity and electrophysiological measures [27,40–42]. In contrast, preliminary analyses show that Akt2 deletion produces selective anxiety and depression-like phenotypes in mice [43], thus suggesting that the Akt genes play differential roles in brain development and function. It is currently unknown whether Akt3 deficiency affects neurobehavioral and cognitive function and what signaling pathways may be involved.

AKT3 is highly expressed in both human and mouse brain [24,25,44], represents up to 50–60% of total AKT in the murine cortex and hippocampus [24] and shows differential prenatal expression patterns, with expression being higher during human fetal brain development [44]. Together these observations suggest a pivotal role for AKT3 in neocortical development and cognitive function. Consistently, numerous genomic studies have suggested a critical role for AKT3 in the developing human central nervous system, specifically in the attainment of normal brain size and intellectual ability. Notably, human germline and somatic mutations and copy-number variations (CNVs) impacting the 1q44 locus and AKT3 have been identified in a range of developmental brain malformation syndromes, including microcephaly [45,46], hemimegalencephaly [47,48] and megalencephalic syndromes [49], all of which are characterized by intellectual disability. Furthermore, studies have consistently shown that Akt3 knockout mice exhibit organ-specific reductions in brain size, with ~20–25% reductions compared to wildtype (WT) mice [24,25].

Here, we examined the role of Akt3 in cognitive function, learning and memory utilizing a novel murine model of Akt3 genetic deficiency and studied the biochemical signaling correlates. Akt3 mice with one (Akt3^-/+^) or zero alleles (Akt3^-/-^) were generated on the C57BL/6 background and compared to WT littermates (Akt3^+/+^). Examination of heterozygote Akt3 mice is an important advance of the current study and provides biologically relevant data on the function of Akt3 in the context of human genetic conditions of Akt3 dysfunction. A comprehensive battery of behavioral and physiological tests relevant to schizophrenia and cognitive dysfunction, chosen to allow dissection of underlying neuroanatomical system deficits were performed along with comprehensive brain biochemical studies of the Akt/mTOR signaling pathway. Our results identify a novel and non-redundant role for Akt3 in prefrontal cortical and hippocampal-mediated cognitive function, identify potential neurobiological mechanisms underlying association of the AKT3 gene with schizophrenia relevant to cortical development, and identify Akt3 as a critical determinant of AKT signaling in the brain.

## Materials and methods

### Subjects and ethics statement

All animal breeding, maintenance and experimental procedures were in accordance with and approved by the National Institute Health Animal Care and Use Committee and followed the National Institutes of Health Using Animals in Intramural Research and the University of Colorado Denver Institutional Animal Care and Use Committee (IACUC). Akt3^-/-^ mice were generated via germ line excision of murine exon 3 (corresponding to nucleotides 209 to 320 of the mouse cDNA), which introduces a frame shift in the open reading frame (first described [24]). Akt3^-/-^ mice were originally generated by Dr. Thomas Ludwig (Columbia University) and Dr. Morris Birnbaum (University of Pennsylvania) and obtained via Material Transfer Agreement with Columbia University. We successfully established Akt3 mutants on the C57BL/6 background (>25 generation of backcrosses) in our laboratory which are maintained as stable colonies. Wild-type (WT, Akt3^+/+^), heterozygous (Het, Akt3^-/+^) and knockout (KO, Akt3^-/-^) mice were generated and included in all assessments and represent littermates derived from multiple independent litters. Mice were group housed (up to 5 animals per cage) and maintained on a 14 hour light/10 hour dark cycle in a climate-controlled animal facility (22±2°C) with food and water *ad libitum*. Animals were sacrificed one week after all behavioral testing by guillotine without anesthesia, according to our approved IACUC method of sacrifice for tissue collection.

### Genotyping

Genotyping was performed by PCR analysis of DNA extracted from tail clips. In brief, DNA extracted by the HOT SHOT method was used as a template for PCR amplification of Akt3 using the following primers: Akt3 forward, Primer 1, (5’-GAT AGG TGG CTT GTG AGT TC-3’); Akt3 wild-type reverse, Primer 2, (5’-CCA GGG TAA GGC CTA AAG CT-3’), which produced a 192kb band from DNAs of wild-type mice. Primers 1 and Akt3 knockout reverse Primer 3, (5’-GCT CAT TCC TCC CAC TCA TGA-3’) produced a 340kb band from DNA isolated from Akt3^-/-^ mice (S1A Fig). Amplification was carried out using Invitrogen Platinum Taq (Thermo Fisher Scientific, PN#10966–018; Grand Island, NY, USA) per the manufacturers protocol, under the following thermal cycling conditions: Initial denaturation: 94°C for 3 min, 40 PCR cycles of: 94°C for 30 sec, 60°C for 30 sec, 72°C for 30 sec, final extension: 72°C for 7 min.

### General physical health

Measures of general health and neurological reflexes were assessed in adult mice, as previously described [50,51]. Mice were observed for measures including posture, fur condition and empty cage behavior to detect irregular behaviors such as excessive grooming, digging, rearing, or stereotypy. Neurological reflexes included trunk curl, limb strength (wire hang test), forepaw reaching, corneal reflex, ear twitch, whisker twitch, and the righting reflex were also examined.

### Behavioral testing

All behavioral tasks were performed on adult male mice, 3 to 5 months old during the light phase. Mice were handled by the experimenter the week preceding testing. To prevent influences of task-related anxiety, animals were split into independent cohorts with the most aversive task performed last. Animals were tested as follows: Cohort 1 (general health, open field, temporal order object recognition, and startle prepulse inhibition); Cohort 2 (social cognition tasks and fear conditioning); Cohort 3 (open field, novel object and fear extinction) and Cohort 4 (open field, object location). All mice were habituated one hour prior to testing. Locomotor activity was performed the day before temporal order object recognition, novel object recognition and object location tasks and served as habituation to the open field. All sessions for open field locomotor activity, sociability, temporal order object recognition, novel object recognition and object location tasks were video recorded with a top mounted CCTV camera (Security Cameras Direct, Luling, TX, USA) using Ethovision XT video tracking system (Noldus; Leesburg, VA, USA).

### Locomotor activity (open field)

The open field test systematically assesses novel environment exploration, general locomotor activity, and anxiety-related emotional behaviors. Baseline locomotor activity was assessed as total distance traveled in an open field experimental arena made of gray polyvinyl (42×42×30cm) under red light luminescence for 60 mins, as previously described [50]. Time spent and distance traveled in the center portion of the arena was assessed as a measure of anxiety.

### Temporal order object recognition memory

The temporal order object recognition task was assessed as previously described [50–53] and as described in detail in S1 Methods. Temporal order measures recency discrimination, defined as a rodent’s ability to differentiate between two familiar objects presented at different intervals and is dependent on information flow between the medial PFC (mPFC), perirhinal (PRH) cortex and hippocampus [52]. Briefly, the task was performed in three, 5-minute sessions, in the same experimental arena used for locomotor activity, under red light luminescence (5±2 lux). If temporal order discrimination memory is intact subjects would exhibit a discrimination ratio > 0. Individual discrimination ratios were calculated as time spent by the animal exploring the object from sample phase 1 minus the time spent with the object from sample phase 2, divided by the total time spent exploring both objects during the test period.

### Novel object recognition

The novel object recognition test was as previously described with small changes [51,53]. Novel object preference requires intact function of the PRH cortex (but not the mPFC or hippocampus [52]). Briefly, the subject was introduced for 10 minutes to two identical objects for the acquisition trial. Two hours later, the mouse was placed back into the same arena with a duplicate of the old object and a new object of the opposite color and shape and allowed to explore for 5 mins. Individual discrimination ratios were calculated as time spent by the animal exploring the novel object minus the time spent with the object from phase 1, divided by the total time spent exploring both objects during the test period. Discrimination ratio > 0 show preference for the novel object.

### Object location memory

Object location was performed to specifically assess hippocampal function and spatial memory [52]. The subject was introduced for 10 minutes to two identical objects for the acquisition trial. One hour later, the mouse was allowed to explore the same two objects for 5 minutes, with one object in its original location and the other counter-opposite to its original position. The object locations were counterbalanced across trials to eliminate the chance of side preference. Individual discrimination ratios were calculated as time spent by the animal exploring the relocated object minus the time spent with the unmoved object from phase 1, divided by the total time spent exploring both objects during the test period. Discrimination ratio > 0 show preference for the moved object and intact spatial memory.

### Acoustic startle and Prepulse Inhibition (PPI)

Startle responses and PPI were measured using SR-Lab Systems (San Diego Instruments; San Diego, CA, USA) as previously described [50,53]. The background level was maintained at 70 dB. The trials consisted of measurements of the startle response to no stimulus (Nulstim), to an auditory startle stimulus (Startle; 40 ms, 120 dB), and of the PPI in response to an acoustic prepulse of 20 ms at 74, 78, 82, 86, and 90 dB.

### Fear conditioning, cued and context

Fear conditioning, dependent on hippocampal and/or amygdala function, was assessed using Pavlovian methodology as previously described [53]. The task was performed using a San Diego Instruments Freeze Monitor System (San Diego Instruments; San Diego, CA, USA) conditioning chamber and is described in detail in S1 Methods.

### Fear extinction

Fear extinction testing was used to assess amygdala depotentiation [54]. Mice were assessed over a total of 3 days. Day 1 (Conditioning Phase), consisted of five trials of five auditory tones (80db) lasting 30 seconds with variable intervals (ranging from 80 to 220 sec) followed by five additional trials of 30 sec tones preceding a 0.5 second foot shock. Day 2 and 3 (Extinction Phases) used identical protocols. On ‘Extinction Phase’ days, mice were exposed to thirty tones lasting 30 seconds each, at variable intervals without any foot shock. Freezing behavior was calculated by the average freezing time (time frozen/allotted time x 100). Normal freezing behavior in mice should progressively decrease over trial days (i.e. % Freezing on Day 1> Day 2> Day 3).

### Social function

Social behavior was tested using methods previously described for assessment of *sociability* and *preference for social novelty* [53,55,56]. Social approach was tested in an automated three-chambered apparatus. Mice used as the novel stimulus target were C57BL/6 matched to the subject mice by sex and age.

### Biochemical assessment of AKT signaling in Akt3-deficient mice

Fresh, frozen mPFC and hippocampus from behaviorally naïve mice was homogenized with a sonicator (Ultrasonic Processor model GE50 Sonics, Newtown, CT, USA) in a lysis buffer containing DTT 1 M (Dithiothreitol, Sigma-Aldrich, St. Louis, MO, USA), protease inhibitor cocktail and T-PER protein Extraction (PN 78510, Thermo Scientific, Grand Island, NY, USA). Thirty or fifty micrograms of protein was resolved per standard Western blotting procedure, as previously described [11,50]. A FluorChemQ image analyzer from Protein Simple (San Jose, CA, USA) was used for Chemiluminescence detection method. The optical density of the protein bands were calculated using Image J software. β-actin was used as a standard loading control for normalization. A full list of primary and secondary antibodies are described in Supplementary material.

### Brain to body weight measurements

Male Akt3 mice were weighed at postnatal day 105 using an Ohaus CS 200 balance (Parsippany, NJ, USA) immediately before sacrifice for brain collection and weighing.

### Quantitative real-time PCR

To quantitatively assess SDCCAG8 expression, total RNA was extracted from PFC and of male Akt3 mice 100 using the TRIzol Extraction method (Qiagen). Quantitative Real-Time RT-PCR was carried out as previously described using the standard curve method [53,57] and TaqMan assay (Hs01104613_m1). For each sample the expression levels of the gene of interest were normalized to the housekeeping gene GAPDH (Mm99999915_g1, Life Technologies).

### Statistical analyses

Data was analyzed using SPSS statistical software (IBM Corp, Armonk, NY, USA v.22). Statistical significance levels were indicated by p<0.05. Fisher LSD posthocs were used to investigate significant findings. General health measures and discrimination ratios for recognition tasks were analyzed with Univariate ANOVA. Repeated Measures ANOVA was used for all other behavioral tasks unless indicated. Linear Regression Analysis was used to examine effects of genotype on quantitative protein traits.

## Results

### Akt3 deficiency results in reduced brain size, with no overall impact on body size or general health

Akt3 mice with one (Akt3^-/+^) or zero (Akt3^-/-^) alleles did not differ from WT (Akt3^+/+^) mice in a comprehensive general health screen (S1 Table), demonstrating physical capability for performing the subsequent battery of behavioral tasks. Consistent with previous reports in Akt3 null mice [24,25], Akt3 deficiency impacted total brain weight. Our data identify that Akt3 deficiency is consistently associated with reduced brain weight, expressed in absolute terms (Main effect of genotype: F (2, 21) = 51.337, p<0.001; posthoc LSD, Akt3^+/+^ vs. Akt3^-/+^ and Akt3^+/+^ vs. Akt3^-/-^, p<0.001; S1b Fig) and as a ratio to body weight (main effect of genotype: F (2, 21) = 49.497, p<0.001; posthoc LSD, Akt3^+/+^ vs. Akt3^-/+^ and Akt3^+/+^ vs. Akt3^-/-^, p<0.001; S1c Fig). In addition, we report a novel allele dose-dependent effect on brain weight, with Akt3 heterozygosity being associated with a 7% reduction in brain weight and null- with a 24% reduction (S1 Fig).

### Baseline locomotor activity is increased in Akt3^-/-^ mice

**Fig 1:**
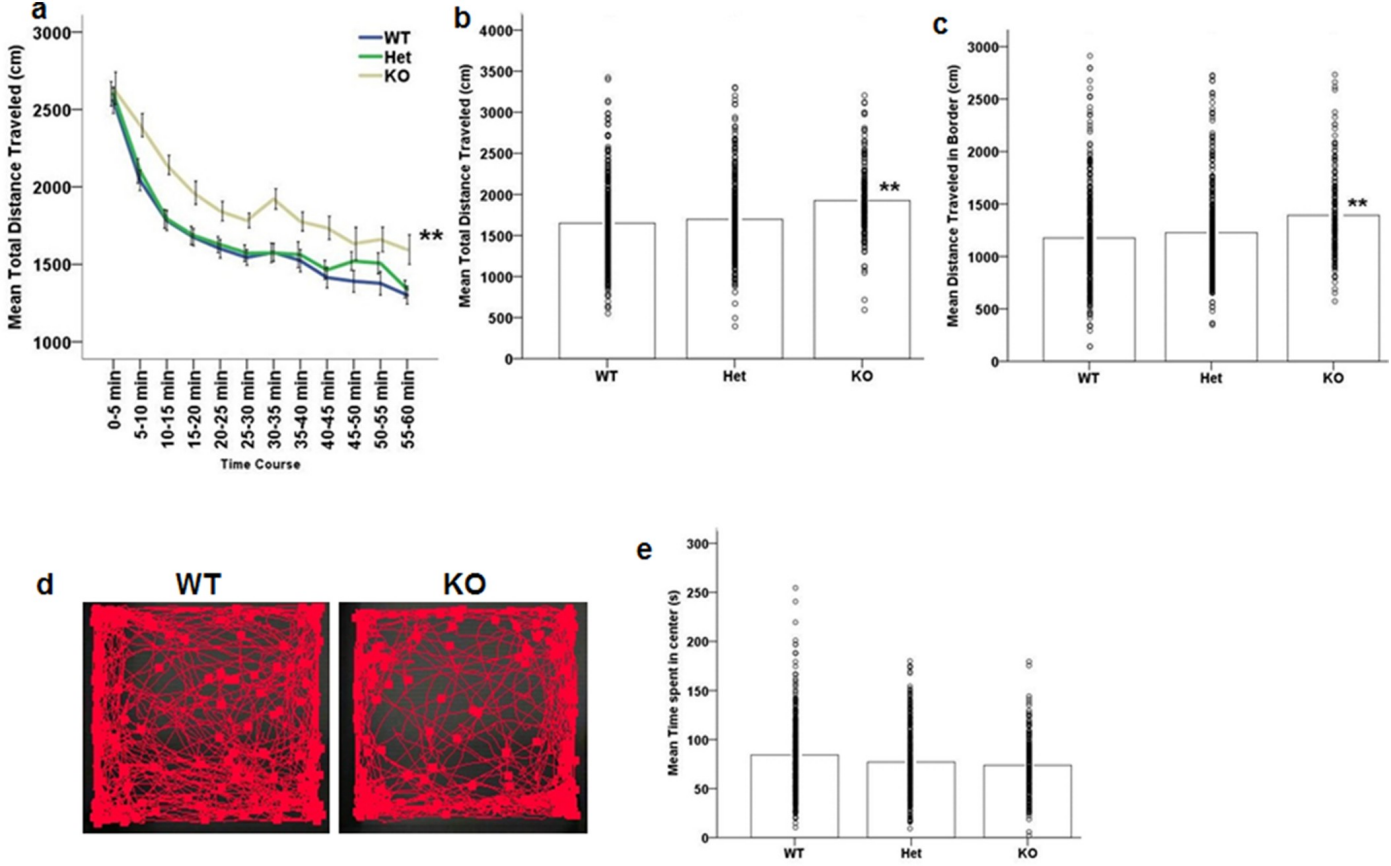
Increased locomotor activity but no anxiety-like phenotype in Akt3^-/-^ mice. (a) Ambulatory distance in 5 min bins displayed by WT and Akt3-deficient mice over the 60 minutes in the open field arena. (b) Akt3^-/-^ (KO) mice display a hyperlocomotive phenotype, as defined by a significant increase in total distance traveled per segment. (c) Hyperlocomotion was accounted for by significant increased distance traveled in the border of the open field apparatus. (d) Representative screen shot of a WT and KO mouse performing the open field task. (e) No effect of genotype on time spent in the center of the apparatus was observed. (a) Data represents mean ± SEM. (b, c, e) Data bars represent the mean, with individual data points representing individual subject measures, n = 29 WT, 28 Het, 15 KO. **p≤0.002 compared to WT.

Repeated measures ANOVA revealed a significant decrease of distance traveled over time during the open field test (main effect of time: F (11, 759) = 136.238, p<0.001; Fig 1a), demonstrating that all mice show similar locomotive habituation. No time x genotype interaction was observed. A significant main effect of genotype was however observed (F (2, 69) = 5.954, p = 0.004) whereby Akt3^-/-^ mice were hyperactive compared to their Akt3^+/+^ and Akt3^-/+^ littermates (posthoc LSD Akt3^+/+^ vs. Akt3^-/-^, p = 0.001; Fig 1b). Further investigation showed that Akt3^-/-^ mice traveled significantly more distance in the border (F (2, 69) = 5.389, p = 0.007; posthoc LSD Akt3^+/+^ vs. Akt3^-/-^, p = 0.002; Fig 1c and 1d). However, there were no differences in distance traveled in the center among genotypes (p>0.1). We also measured time spent in the center of the apparatus to assess if Akt3 deficiency is associated with anxiety-like phenotypes. Our data indicate no effect of genotype on time spent in center (p>0.1, Fig 1e). These data reveal that genetic loss of Akt3 in the mouse results in hyperlocomotive behaviors similar to those observed in models of psychostimulant-mediated effects on increased dopaminergic function and suggestive of a novel role of Akt3 in dopaminergic signaling.

### Deficits of temporal order object discrimination memory, object location memory, but not novel object preference in Akt3-deficient mice

**Fig 2:**
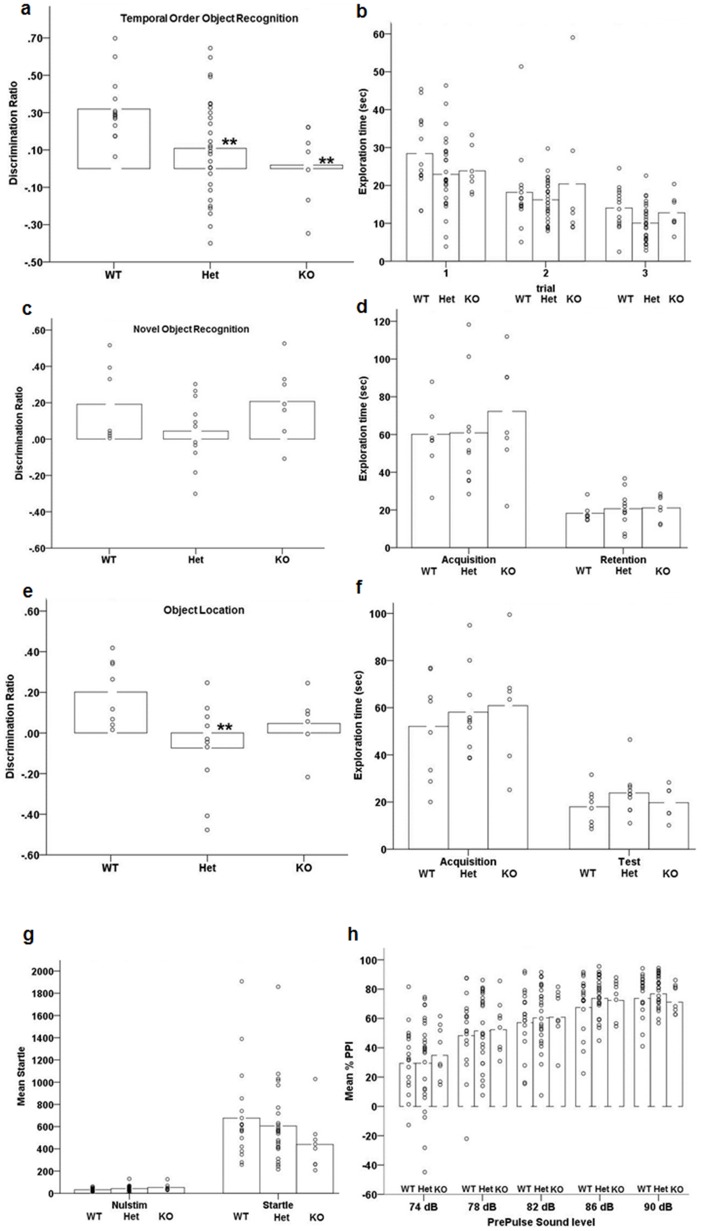
Impaired temporal order object discrimination memory and spatial location memory in Akt3-deficient mice. (a) Discrimination ratio displayed by Akt3-deficient mice during the 5 min test phase (sample 3) of the temporal order object recognition task. (** p≤0.01 by posthoc LSD; n = 14 WT, 28 Het, 7 KO). (b) Total time spent exploring the objects presented during the 5-min sample phases 1, 2 and 3. (c) Discrimination ratio for the novel object by Akt3-deficient mice during the novel object test (n = 9 WT, 11 Het, 7 KO). (d) Time spent exploring the two objects during the 10 min acquisition session and the 5 min retention session of the novel object recognition test. (e) Discrimination ratio for the moved object over the same-placed object (**p≤0.01; n = 9 WT, 10 Het, 6 KO). (f) Time spent exploring the two objects during the 10 min acquisition and the 5 min test session of the same object location test. (g) Absence of differences in baseline movement (Nulstim) or Startle responses in the pre-pulse inhibition test in Akt3-deficient mice. (n = 18 WT, 26 Het, 8 KO). (h) Percentage prepulse inhibition of the acoustic startle response displayed by the same mice after the presentation of prepulse (n = 18 WT, 26 Het, 8 KO). Data bars represent the mean, with individual data points representing individual subject measures.

To determine whether Akt3 is involved in cognitive performance we tested Akt3 mice in a series of recognition memory tasks, including temporal order discrimination memory, novel object preference and the object location test. The combination of these tests can be used to examine the neuroanatomical components of dysfunction, with each task involving specific regions involved in components of the tests in question. Temporal order object recognition memory is critically dependent on the mPFC and integrated information flow with the perirhinal cortex and hippocampus [52]. Novel object preference requires intact function of the PRH cortex (but not the mPFC and the hippocampus), while object location is critically dependent on hippocampal function for spatial memory [52]. ANOVA revealed a significant effect of genotype on discrimination performance during the temporal order object recognition task (F (2, 46) = 4.805, p = 0.013; Fig 2a), driven by significant deficits in the Akt3-deficient mice (posthoc LSD, Akt3^+/+^ vs. Akt3^-/+^, p = 0.011; Akt3^+/+^ vs. Akt3^-/-^, p = 0.010). Akt3-deficient mice did not differ in the amount of exploration in the different phases of the task (Fig 2b; p>0.1).

Next, we tested a separate naïve cohort of mice in the novel object preference task. ANOVA revealed no effect of genotype on novel object discrimination (p>0.18; Fig 2c), with all mice significantly exploring the novel object more than the familiar during the test phase (main effect of trial; F (1, 24) = 102.298, p<0.001; Fig 2d). Exploration time within the different phases of the task (acquisition and retention) did not differ between genotype groups (p>0.6, Fig 2d). These findings suggest intact function of the perirhinal cortex in the context of Akt3 deficiency [52].

Finally, a third cohort of naïve mice were assessed in the object location task to examine spatial memory, dependent on intact hippocampal function [52]. We observed a significant effect of genotype on spatial memory (F (2, 21) = 3.755, p = 0.02). WT mice showed a positive discrimination ratio for the relocated object over the originally placed object (Fig 2e), which was significantly impaired in Akt3-deficient mice (posthoc LSD, Akt3^+/+^ vs. Akt3^-/+^, p = 0.006; Akt3^+/+^ vs. Akt3^-/-^, p = 0.14). Akt3-deficient mice did not differ in their extent of exploration of the objects during the acquisition and test phases of the task (p>0.2; Fig 2f). Together, these observations demonstrate deficits of prefrontal cortical and/or hippocampal-mediated cognitive function in the context of Akt3-deficiency.

### Sensorimotor gating is normal in Akt3-deficient mice

Prepulse Inhibition (PPI) evaluates sensorimotor gating in the pre-pulse inhibition of the acoustic startle response and is a deficit observed commonly in neurodevelopmental disorders, including schizophrenia [58]. One-way ANOVA indicates no significant differences in nulstim (p>0.06) or startle (p>0.3) between genotypes (Fig 2g). Significant increases of inhibition across prepulse were observed in all groups (F (4, 196) = 78.345, p<0.001; Fig 2h), with no genotype effect or genotype x prepulse interaction (p>0.8).

### Learned fear conditioning and fear extinction are intact in Akt3-deficient mice

**Fig 3:**
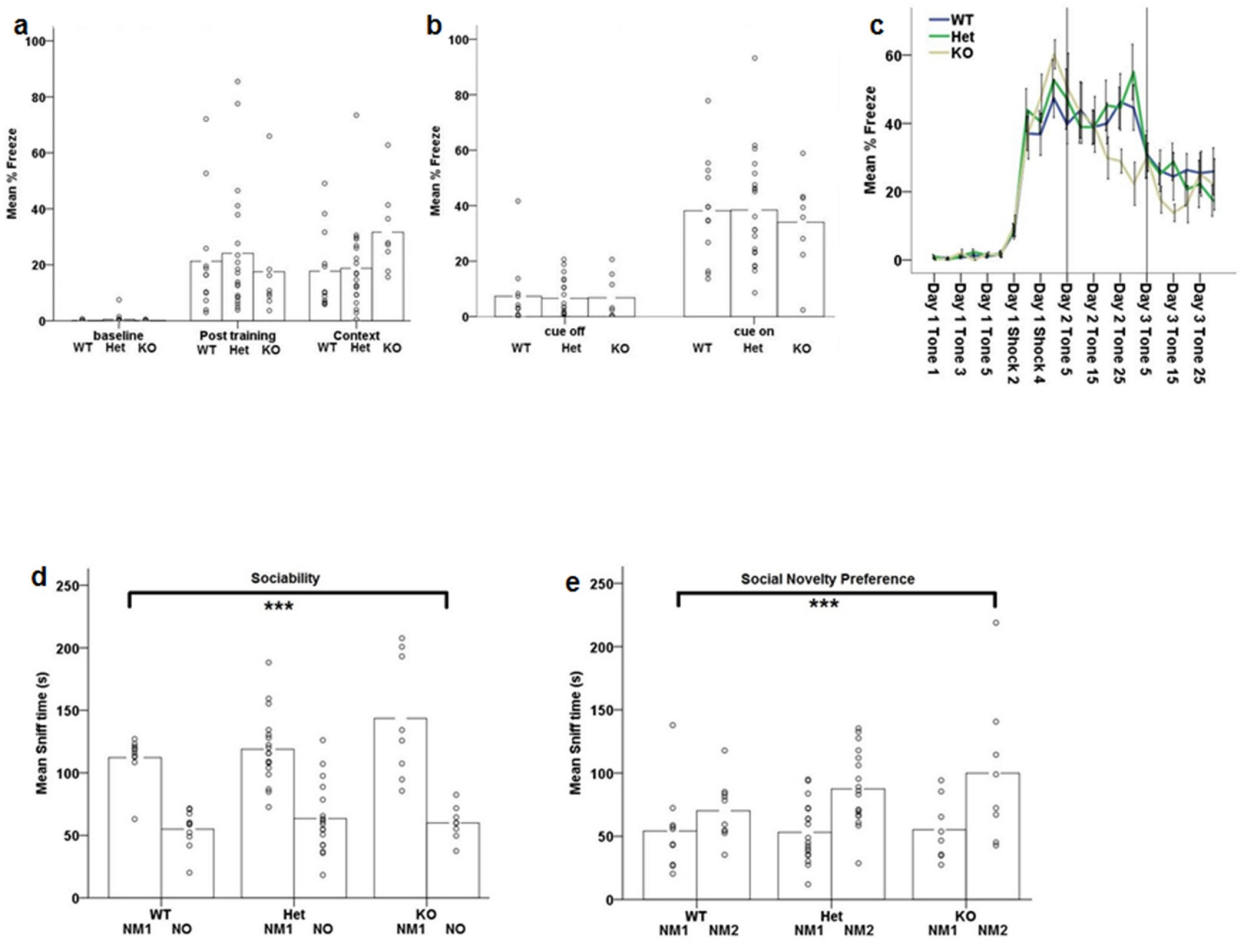
Akt3-deficiency does not impact fear learning and extinction, or social behavior. (a) Freezing behavior prior (baseline), subsequent (post training), and 24 hours later (context 24hr) after the cue-shock pairings. All genotypes increased freezing subsequent to the CS-US pairings and genotypes did not differ during the testing of contextual conditioning (n = 12 WT, 20 Het, 8 KO). (b) Freezing behavior during testing for cued conditioning in the altered context without (CS off) and plus the auditory cue (CS on; n = 12 WT, 20 Het, 8 KO). (c) Fear extinction after repeated exposure to the auditory cue (CS alone) shows no effect of genotype of genotype x day interaction (n = 13 WT, 13 Het, 9 KO) (d) Time spent sniffing the novel mouse during the 10-min test for sociability was greater than time spent sniffing the novel object for all mice (n = 10 WT, 18 Het, 8 KO). (e) Time spent sniffing the novel mouse 2 was greater than time spent sniffing novel mouse 1 (familiar) during the 10-min test for social novelty was greater for all mice (n = 10 WT, 18 Het, 8 KO). ***p≤0.001 main effect of chamber. (a,b,d,e) Data bars represent the mean, with individual data points representing individual subject measures. (c) Data represents mean ± SEM.

Using Pavlovian fear conditioning, we evaluated Akt3’s role in learning and memory mediated via hippocampal and amygdala neural circuitry. Notably, the hippocampus is a key mediator of the acquisition and expression of conditioned fear to a particular context, while the acquisition and extinction of auditory cued fear memories relies on the amygdala [59]. During the context fear test, we observed significant freezing behavior during the post training (acquisition) and contextual fear phases (main effect of phase: F (2, 74) = 35.972, p≤0.001; Fig 3a), with no significant genotype effects (p>0.8). At baseline, freezing was equal to zero in all groups. Re-exposure to the same context 24hrs, without cue, elicited a similar pattern of % freezing to that observed post training in all genotypes (Fig 3a). These data indicate that Akt3 does not impact fear learning and memory. Similarly, during the cued fear test all mice displayed normal cued conditioned freezing, revealed by a significant increase in freezing to the auditory cue (F(1, 37) = 118.634, p<0.001; Fig 3b) with no significant effect of genotype or interactions (p>0.2). To further assess synaptic efficacy within the amygdala, we used auditory fear extinction to assess depotentiation, the reversal of the learned condition-induced potentiation of the fear response [54]. All mice displayed a significant decrease of freezing over trials (F (21, 672) = 41.917, p<0.001; Fig 3c) with no effect of genotype (p>0.1).

### Akt3 deficiency does not impact social functioning

Abnormalities of social cognition are present in various neurodevelopmental disorders, including schizophrenia and autism [60–62]. To assess if Akt3 impacts social interaction behaviors, we examined sociability and social novelty utilizing the three-chamber sociability test as previously described in detail [50,53]. Analysis of sociability revealed that all mice exhibited a main chamber preference as assessed by exploration measured as sniff time (main effect of chamber: F (1, 33) = 121.336, p<0.001; Fig 3d) and time spent in the chamber containing the novel mouse vs. the novel object (F(1, 33) = 30.920, p<0.001), with no effect of genotype or interactions observed (p>0.2). For social novelty, all mice displayed significant preference for interaction with the novel mouse (2) vs. the familiar mouse (mouse 1) (main effect of chamber measured as exploration via sniff time: (F (1, 33) = 15.724, p<0.001; Fig 3e)), independent of genotype (p>0.3). These observations suggest that social functioning is preserved in the context of Akt3 deficiency.

### Biochemical signaling in Akt3-deficient mice

**Fig 4:**
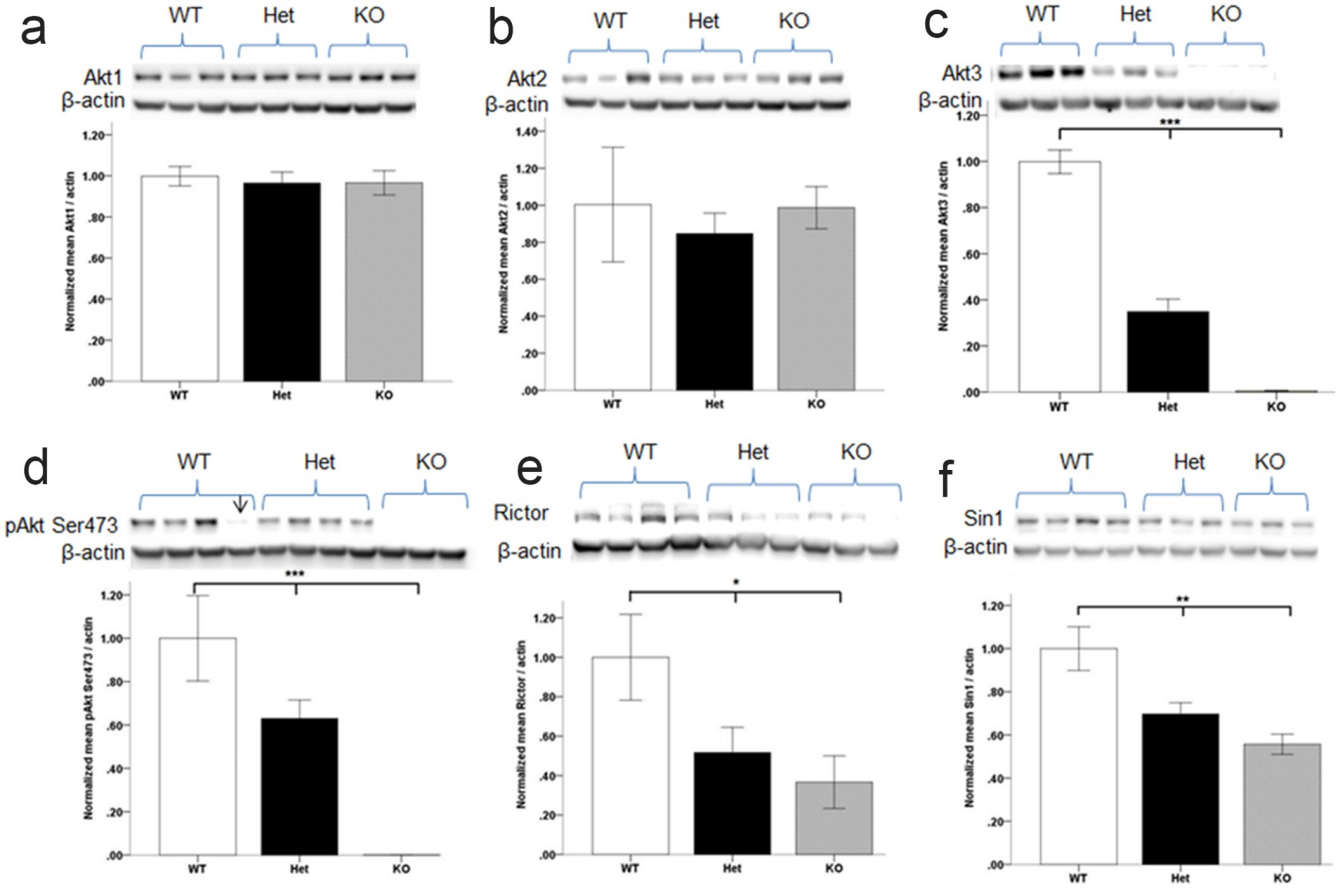
Biochemical changes in the mPFC of Akt3-deficient mice as determined by Western blot. Absence of compensatory changes in Akt1 (a) or Akt2 (b) protein levels in Akt3-deficient mice. (c) Akt3 protein is significantly reduced in Akt3 (^-/+^) Het mice and absent in (^-/-^) KO mice. (d) Reduced pAKT Ser^473^ in Akt-deficient mice. (e) Total Rictor and Sin 1 (f) protein was decreased in Akt3 mutant mice. * p≤0.05, **p≤0.01, ***p≤0.001. Quantitative data derived from n = 3–6 per genotype. Data represents mean ± SEM. Representative Western blots are shown. Arrow denotes excluded sample due to gel bubble.

We next examined expression and activity of select proteins in the AKT/mTOR signaling network in the mPFC of Akt3-deficient mice. To address the issue of isoform redundancy and biological compensation, we first measured total Akt1 and Akt2. Linear regression analyses revealed no differences in levels of Akt1 (p = 0.8; Fig 4a) or Akt2 (p = 0.8; Fig 4b) protein, consistent with previous findings in Akt3 null mice [24, 25]. Our findings also demonstrate that hemizygous deletion of Akt3 does not result in changes in the level of Akt1 (Fig 4a) or Akt2 (Fig 4b). Linear regression revealed that 91% of the variance in Akt3 protein was explained by genotype (Model: F (1,16) = 160.30, p<0.0001; Genotype; β = −.0.95; t = −12.66; p<0.0001), with decreased levels in Akt3^-/+^ mice and absence of detection in Akt3^-/-^ mice (Fig 4c). The activation status of Akt, as measured by phosphorylation of the pAkt Ser^473^ site, a conserved site across all three isotypes, was examined in Akt3-deficient mice. Linear regression revealed that 78% of the variance in pAKT Ser^473^ was explained by genotype (Model: F (1,9) = 33.33, p<0.001; Genotype; β = −0.89; t = −5.77; p<0.001), with decreased levels in Akt3^-/+^ mice and absence of detection in Akt3^-/-^ mice (Fig 4d). Remarkably similar findings were observed in the hippocampus (Model: F (1,9) = 71.01, p<0.001; Genotype; β = −0.94; t = −8.4; p<0.001; S2 Fig). Phosphorylation of Ser^473^ is mediated by the PDK2/Rictor/Sin1-mTOR complex, mTORC2. Significant effects of genotype were observed on levels of Rictor and Sin1, critical components of mTORC2 (Rictor; Model: F(1, 9) = 6.96, p = 0.03; Genotype; β = −0.68; t = −2.63; p = 0.03; Sin1; Model: F(1, 9) = 16.88, p = 0.003; Genotype; β = −0.82; t = −4.1; p = 0.003), with reduced levels observed in the context of Akt3 deficiency (Fig 4e and 4f). Given the evidence for overall reduced activity of Akt in the context of Akt3 deletion, not compensated for by changes in total Akt1 or Akt2, we predicted altered expression/activation of downstream targets of Akt. The phosphorylation levels and total levels of glycogen synthase kinase 3 (GSK3β), pGSK3β/GSK3β were unaltered and proteins indicative of mTORC1 signaling were not altered in Akt3 mice (total mTOR; phosphorylation of mTOR at (Ser2448) and p70S6K). No changes were observed in the total levels of PSD95, PI3K, p110δ (PI3Kδ), AMPA receptor (GluR 2/3/4), NMDA receptor (NMDAR1) or PDK1. No effects of genotype were observed for expression levels of the SDCCAG8 gene (S3 Fig). Expression of the housekeeping gene and loading control, β-actin did not significantly differ across genotypes (Fig 4; p>0.9)

## Discussion

Akt3 is the predominant Akt isotype in the adult brain [24,25], transcription is enriched in human fetal neocortex [44] and haploinsuffiency in humans is linked to cortical malformations, including microcephaly and cognitive dysfunction [45,46,63]. Despite this evidence, little is known about the neurobiological role and signaling specificity of AKT3. In the present study, we utilized Akt3 hemizygous and nullizygous mice in a pure C57BL/6J background and provide evidence for a causal relationship between Akt3 levels and the attainment of normal brain size, extending prior findings [24,25] to show that heterozygous reduction of Akt3 is associated with selective, subtle and dose-dependent decreases in brain size (7%). Moreover, we demonstrate that Akt3 is critical for neurocognitive performance linked to prefrontal cortical-hippocampal circuitry and that these changes are associated with a dramatic reduction of activated AKT (as measured by AKT Ser^473^ phosphorylation), deficits not accounted for by compensation of Akt1 or Akt2 levels. Together, these data implicate an indispensable and non-redundant role for Akt3 in brain development and cognitive function and identify neurobiological mechanisms of relevance to schizophrenia.

Contemporary psychiatric genetic studies have identified polymorphic variation in the AKT3 gene, including a putative functional SNP (rs14403) in the 3 untranslated region (UTR) of transcript NM_005465, as genome-wide associated to schizophrenia [5,8]. While the molecular mechanisms of risk remain to be determined, it is noteworthy in the context of our findings, that subtle whole brain- and cortical grey matter volume reductions have been observed in patients with schizophrenia [64–66], with meta-analyses of postmortem brain and structural imaging studies showing 2–3% global brain volume reductions, with larger reductions in the PFC and temporal lobe [64,66,67]. Although there is considerable debate about the origins of structural brain changes in schizophrenia, including secondary effects of antipsychotic drugs, drugs of abuse and illness [68], many changes are present to a lesser degree in first-episode patients [68–70]; and show evidence of heritability in patients and siblings [66,71]. Recent large-scale imaging genetic studies also provide evidence of a shared genetic basis of several neuroanatomical phenotypes and schizophrenia risk [72]. Evidence of association to genes which control key biological processes related to structural brain development, therefore implicate a potential neurobiological mechanism of risk. Consistently, a new genome-wide association study of 32,438 adults shows that variants determining human intracranial volume and related cognitive function are enriched in genes for PI3K-AKT signaling, including AKT3 [73]. Interestingly, rs7538011, an intronic variant associated with human intracranial volume [73], is in linkage-disequilibrium (LD) with the schizophrenia 3 UTR risk variant, rs14403 (D^’^ 0.92; R^2^ 0.52) and has membership in one of the top pathways showing cross-disorder association to psychiatric risk [6].

Previous examination of Akt3 null mice shows that reduced brain size ([24] and confirmed in this study) is apparent at birth and histologically defined by a reduction in cortical cell size and density [24], potentially consistent with Akt3’s reported role in axonal and dendritic development [74,75]. Notably, similar cytoarchitectural changes have been reported in postmortem brain of patients with schizophrenia [76–80] and in conjunction with evidence for genetic association to biological pathways that regulate axonal, dendritic, and postsynaptic development [6], suggest that at least some neuropathological changes may be genetically determined, neurodevelopmental in origin, and potentially related to alterations in AKT signaling.

Mammalian brain size, in particular the magnitude of the cerebral cortex, is a significant determinant of cognitive ability [81,82] and several disorders of neurodevelopment, including schizophrenia are characterized by cognitive dysfunction [83]. Behavioral analysis revealed that genetic reduction of Akt3 produces selective deficits in mPFC and/or hippocampal mediated neurocognitive function, as evidenced by discrimination impairments of temporal order object recognition memory and spatial memory. Such findings are similar to temporal and spatial memory impairments observed in patients with schizophrenia [84–86] and consistent with abnormalities of prefrontal cortical function in the disorder [1,3, 87–89]. These novel results identify Akt3 as a critical determinant of prefrontal cortical and hippocampal development, not previously appreciated. Indeed, Akt3 is more transcriptionally active in cortex and hippocampus compared to other brain regions, [24,45] further supporting regional biological importance. Study of the effects of schizophrenia-risk associated AKT3 polymorphisms (as previously done for AKT1 [34,35,37]) on neuroanatomical structure and neurocognitive function in normal human subjects, is warranted.

The results presented here also highlight a key role for Akt3 in the expression of dopamine-mediated behaviors, with Akt3 null mice exhibiting a generalized increase in baseline locomotor activity in the open field. These findings are in contrast to observations in Akt2 null mice, whereby decreased ambulatory behavior was observed in the context of an anxiety like phenotype [43], and Akt1 null mice where no differences in locomotion were observed [90]. D2, dopamine receptors (the established target of antipsychotic drugs) are critical regulators of AKT signaling in brain [91,92], mediated via the β-arrestin 2/PP2A complex [93]; whereby dopamine-mediated signaling, and D2 receptor antagonism, result in inactivation and activation of AKT, respectively. [27, 91,92]. Which AKT (1, 2 or 3) mediates these effects is still unclear, although some evidence suggests a potential role for Akt1 [27]. Our data in this context implicate a novel role for Akt3 in dopamine neurotransmission and highlight more targeted approaches towards Akt3 for targeted neuroleptic drug development.

Biochemically, phosphorylation of Ser^473^ was dramatically diminished in the brain of Akt3 mutant mice, with Akt3^-/+^ mice showing 40% reduction and Akt3^-/-^ showing complete abolishment, compared to WT. These effects were not accompanied by changes in total Akt1 or Akt2, supporting previous observations in Akt3 nullizygous mice [24,25], and suggest that Akt3 is a primary determinant of Ser^473^ activity in brain and a critical Akt isotype that does not exhibit redundancy. Phosphorylation status of Ser^473^ is used as an endogenous functional readout of mTORC2 kinase activity and in agreement, while the loss of Akt3 did not affect levels of mTOR, it did significantly abrogate levels of Rictor and Sin1, critical components of the PDK2/mTORC2 complex [16,94]. Importantly, all sites indicative of mTORC1 activation [94,95], including phospho-mTOR (Ser2448) and p70S6K were unaltered in Akt3 mice. These results suggest an mTORC2-dependant mechanism is responsible for the microcephaly and behavioral impairments in Akt3 mutant mice. Remarkably, ablation of the mTORC2 component, Rictor [94], produces dramatically reduced Akt Ser^473^ activation, microcephaly and alterations in neuronal morphology and function, without impact on mTORC1 signaling. Consistent results are also observed for Sin1 ablation [16]. These findings are strikingly reminiscent of Akt3 deletion and suggest a previously unappreciated feedback loop between Akt3 and mTORC2 and show that in brain, Akt3 may be the primary target for mTORC2 activity. The related mechanisms, however, remain to be determined. It is noteworthy that attenuated AKT activity and phosphorylated AKT Ser^473^ [39,40,96] is observed in patient brain and peripheral cells, suggesting that impaired PI3K-AKT signaling is relevant to etiopathogenesis of schizophrenia. Our data in mouse provide support for this hypothesis and demonstrate that reductions in Akt3 can result in attenuated brain AKT activity and phenotypic outcomes similar to observations in schizophrenia. Since cognitive deficits remain a significant therapeutic problem in schizophrenia, these findings highlight the AKT signaling pathway and Akt3 specifically, as a biologically relevant therapeutic target.

Finally, one potential shortcoming of in-vivo gene-targeting technologies is the issue of disrupting flanking genes [97]. This is particularly important for study of Akt3 function, given that SDCCAG8, a gene also implicated in schizophrenia [8, 98], flanks Akt3 both in humans and mouse and shares regulatory regions. We measured brain SDCCAG8 expression in Akt3 mice and show no differential expression based on genotype. This finding is important as it demonstrates that phenotypes observed in Akt3 mutant mice are the direct result of Akt3 disruption.

## Conclusion

In summary, our study validates the essential role of Akt3 in the attainment of normal brain size and reveals a critical role for Akt3 in prefrontal cortical-hippocampal mediated cognitive function relevant to genetic risk for schizophrenia. Furthermore, we identify a central, non-redundant role for Akt3 in governing brain Akt signalling and identify Akt3 as potential novel pharmacological target for neuroleptic treatment development.

## Supporting information

S1 Table Physical characteristics, motoric abilities, sensory reflexes and empty cage behavior of male Akt3 mice. No general health abnormalities were observed. Values represent percentage or mean ± SEM. n = 14 WT, 21 Het, 4 KO.
(DOCX)

S1 Fig Reduction of brain weight in Akt3-deficient mice. (a) PCR genotyping from wild-type (Akt3^+/+^), Akt3 knockout (Akt3^-/-^) mice, and Akt3 heterozygous (Akt3^-/+^) genomic DNA. Primers 1 and 2 generate a 192 kb band from wild-type DNA, while primers 1 and 3 generate a 340 kb band when Akt3 is the targeted allele. (b) Representative average brain weights for Akt3 male mice. Akt3 Het and KO mice show significant decreases in brain weight compared to littermate controls, indicating an allele dose impact on brain weight with a 7% and 24% reduction respectively. (c) There was a significant decrease in both Het and KO mice brain to body ratios when compared to WT littermate male Akt3 mice. n = 10 WT, 7 Het, 7 KO. ***p≤0.001 compared to WT; ### p≤0.001 compared to Het.
(TIF)

S2 Fig Reduced pAKT Ser473 in hippocampus of Akt-deficient mice. ***p≤0.001. Quantitative data derived from n = 3 per genotype. Data represents mean ± SEM.
(JPG)

S3 Fig Akt3 mutant mice do exhibit alterations in SDCCAG8 gene expression in the mPFC. n = 6 per genotype.
(TIF)

S1 Methods Detailed description of behavioral and biochemical methods and reagents used for experiments included in this manuscript. (PDF)
