# Supplementary material for: PKBγ/AKT3 loss-of-function causes learning and memory deficits and deregulation of AKT/mTORC2 signaling: Relevance for schizophrenia
Source: PLoS One. 2017 May 3;12(5):e0175993. doi: 10.1371/journal.pone.0175993 (PMC5414975; doi:10.1371/journal.pone.0175993)
Supplement: S1 Table — No general health abnormalities were observed. Values represent percentage or mean ± SEM. n = 14 WT, 21 Het, 4 KO. (DOCX) [file pone.0175993.s001.docx]

| **General Health** | **WILDTYPE** | **HETROZYGOTE** | **KNOCKOUT** |
| --- | --- | --- | --- |
| Body weight (g) | **25.07 ± 0.48** | **27.02 ± 0.47** | **27.5 ± 1.04** |
| Poor coat condition (%) | **0** | **0** | **0** |
| Bald Patches (%) | **0** | **0** | **0** |
| Missing whiskers (%) | **0** | **0** | **0** |
| Piloerection (%) | **0** | **0** | **0** |
| Body tone (% of good) | **100** | **100** | **100** |
| Limb tone (% of good) | **100** | **100** | **100** |
| Physical abnormalities (%) | **0** | **0** | **0** |
| **Motoric abilities** |  |  |  |
| Trunk curl (%) | **100** | **100** | **100** |
| Forepaw reaching (%) | **100** | **100** | **100** |
| Wire hang (sec) | **60±0.00** | **54.66±2.49** | **60.0±0.00** |
| Positional passivity (%) | **0** | **0** | **0** |
| **Reflexes (% of mice normal)** |  |  |  |
| Righting reflex (%) | **100** | **100** | **100** |
| Corneal (%) | **100** | **100** | **100** |
| Ear twitch (%) | **100** | **100** | **100** |
| Whisker twitch (%) | **100** | **100** | **100** |
| **Reactivity** |  |  |  |
| To handling (3-point scale) | **2.0±0.0** | **2.0±0.0** | **2.0±0.0** |
| Petting escape (%) | **0** | **0** | **0** |
| **Empty cage behavior** |  |  |  |
| Transfer freezing (%) | **0** | **0** | **0** |
| Wild running (%) | **0** | **0** | **0** |
| Exploration (3-point scale) | **2.0±0.0** | **2.0±0.0** | **2.0±0.0** |
| Grooming (sec) | **5.61 ± 1.07** | **5.74 ± 0.76** | **4.61 ± 0.25** |
| Grooming (events) | **5.79 ± 0.87** | **6.29 ± 1.22** | **4±0.58** |
| Rearing (events) | **22.43 ± 2.16** | **26.27 ± 1.60** | **32.5±4.77** |
| Digging (events) | **4.36 ± 0.75** | **4.27 ± 0.59** | **5.75 ± 2.18** |
