# Supplementary figures and images for: PKBγ/AKT3 loss-of-function causes learning and memory deficits and deregulation of AKT/mTORC2 signaling: Relevance for schizophrenia
Source: PLoS One. 2017 May 3;12(5):e0175993. doi: 10.1371/journal.pone.0175993 (PMC5414975; doi:10.1371/journal.pone.0175993)

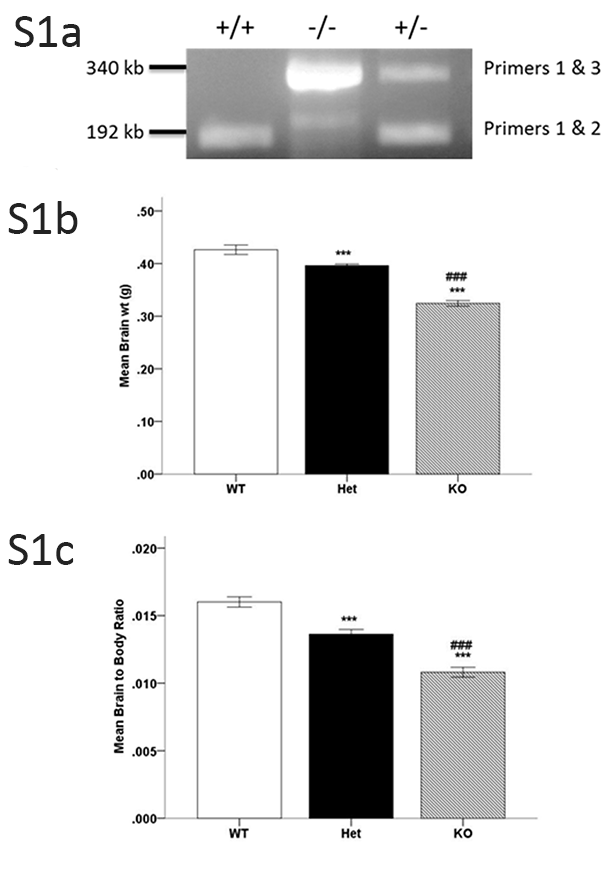

Supplement: S1 Fig — (a) PCR genotyping from wild-type (Akt3+/+), Akt3 knockout (Akt3-/-) mice, and Akt3 heterozygous (Akt3-/+) genomic DNA. Primers 1 and 2 generate a 192 kb band from wild-type DNA, while primers 1 and 3 generate a 340 kb band when Akt3 is the targeted allele. (b) Representative average brain weights for Akt3 male mice. Akt3 Het and KO mice show significant decreases in brain weight compared to littermate controls, indicating an allele dose impact on brain weight with a 7% and 24% reduction respectively. (c) There was a significant decrease in both Het and KO mice brain to body ratios when compared to WT littermate male Akt3 mice. n = 10 WT, 7 Het, 7 KO. ***p≤0.001 compared to WT; ### p≤0.001 compared to Het. (TIF) [file pone.0175993.s002.tif]

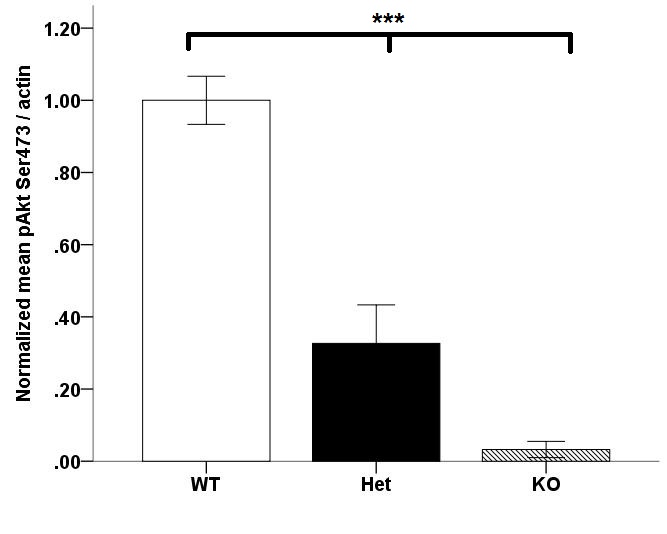

Supplement: S2 Fig — ***p≤0.001. Quantitative data derived from n = 3 per genotype. Data represents mean ± SEM. (JPG) [file pone.0175993.s003.jpg]

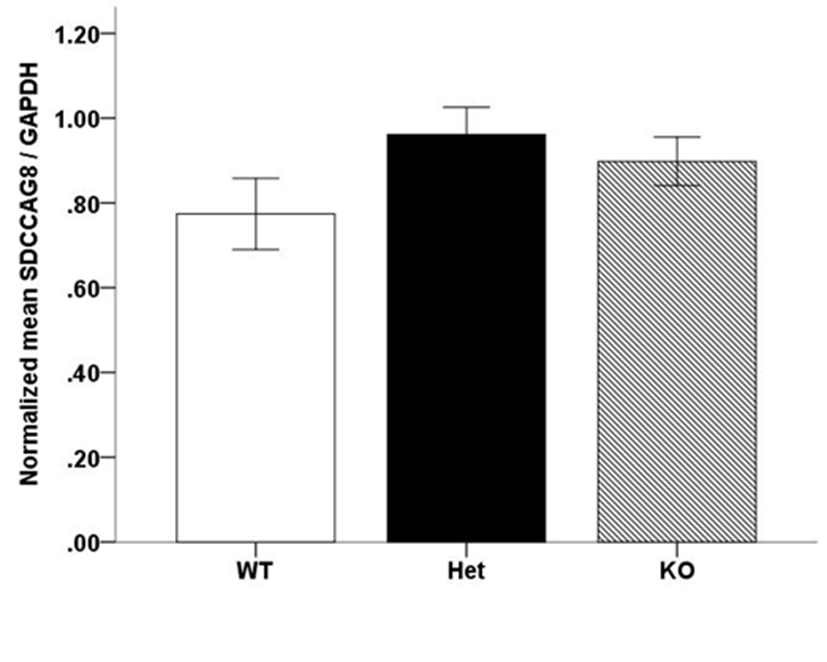

Supplement: S3 Fig — n = 6 per genotype. (TIF) [file pone.0175993.s004.tif]
