# Supplementary material for: PKBγ/AKT3 loss-of-function causes learning and memory deficits and deregulation of AKT/mTORC2 signaling: Relevance for schizophrenia
Source: PLoS One. 2017 May 3;12(5):e0175993. doi: 10.1371/journal.pone.0175993 (PMC5414975; doi:10.1371/journal.pone.0175993)
Supplement: S1 Methods — (PDF) [file pone.0175993.s005.pdf]

## Supplemental Methods

**Temporal Order Object Recognition:** One hour spanned between the first and second session, and a three hours between the second and third sessions. During the first session, mice are placed in the apparatus with two identical objects and allowed to explore. During the second session mice are placed with two different identical objects and allowed to explore. During the third session ('test phase'), mice were placed with duplicates of each object from the previous two sessions and allowed to explore. If temporal order memory is intact, the subjects will spend more time exploring the object from session 1 (least familiar) compared with the object from session 2 (most familiar). Discrimination between objects was calculated using a ratio that takes into account individual differences in the total amount of exploration and calculated as (time spent by each animal exploring the object from sample phase 1 – time spent exploring the object from sample phase 2 / by the total time spent exploring both objects during the test period). If temporal order discrimination memory is intact, subjects will spend more time exploring the object from session 1 (i.e. less familiar object) than the object from session 2 (i.e., the newer object). If temporal order discrimination memory is intact subjects will have a discrimination ratio >.0. The objects consisted of two rectangular boxes (3x3x6 cm), or two laboratory flasks (4x6 cm), each either black or white. The positions of the objects in the test and the objects used in the different phases were counterbalanced between the genotypes. Time spent exploring each object was subsequently scored from the videotapes as the number of seconds when each subject was facing the object and ≤1 cm away. Any subjects that failed to complete a minimum of 2 seconds exploration in the experimental phases were excluded from the analysis.

**Fear conditioning, cued and context:** The conditioned stimulus (CS) was an 80dB tone for 30 seconds and the unconditioned stimulus (US) was a 0.5mA, 2 second scrambled foot shock delivered to the floor grid. For conditioning training on day 1, a 2 minute baseline measurement of freezing in the conditioning chamber was assessed (baseline). Freezing was assessed during three, 30 second conditioning trials with 90-second inter-trial delays followed by 2 minutes post conditioning (post training). On day 2, freezing was measured upon the return to the original conditions of the box and monitored for 5 minutes (context). Two hours after contextual fear testing, subjects were retested to assess cued conditioning in a novel environment created using thick, opaque plastic to line the wire floor grid and walls and a 1% vanilla extract solution for a novel scent. Freezing was monitored for 2 minutes (cue off), during exposure to the CS (cue on), followed by an additional 2 minutes. We are able to disentangle the functionality of the amygdala via the cued (auditory CS) portion of the Fear Conditioning task by changing the context of the box to negate the role of the hippocampus, which is crucial for contextual spatial memory.

**Biochemical assessment of AKT signaling in Akt3-deficient mice:** The following primary antibodies were diluted in 5% milk in TBST (Tris-Buffered Saline 1% Tween 20): anti-AKT-3 (1:1500, MAB #05-780), anti-AKT-2 (1:500, SC #5270), anti-AKT-1 (1:1000, CS #2938), anti-pAKTser473 (1:1000, CS #9271), anti- pAKTthr308 (1:1000, CS #2965), anti-mTOR (1:1000, CS #2972), anti- pmTOR-Ser2448 (1:1000, CS #2971), anti-S6 (1:1000, CS #2317), anti-p70S6K (1:500, CS #5364), anti-PI3Kinase p110δ (1:500, SC #55589), anti-NMDAR1 (1:1000, CS#5704), anti-AMPA Receptor (GluR 2/3/4) (1:1000, CS #2460), anti-pPDK1 (1:1000, CS #3061), anti-PDK1 (1:1000, CS #3062), anti-phospho p70S6 (1:1000, CS #9205), anti-p70S6 (1:1000, CS #9202), anti-pGSK3β (1:1000, CS9323), anti-GSK3β (1:2500, BD Transduction #610201), anti-PSD95 (1:1500, MAB-N68), and anti-β-actin-peroxidase (1:10,000, Sigma Aldrich #A3854). HRP-conjugated secondary antibodies (Santa Cruz Biotechnology, Dallas, TX, USA) against mouse and rabbit were used. Chemiluminescence was developed using a FLuorChem Q imaging system (Protein Simple, San Jose, CA, USA). Image J software (Wayne Rasband, NIH, Bethesda, MD, USA) was used for calculating optical density. Phosphorylated proteins were normalized to the respective total protein levels and total proteins were normalized to β-actin for analyses. Abbreviations, MAB: EMD Millipore (Billerica, MA, USA); CS: Cell Signaling Technologies (Danvers, MA, USA); SC: Santa Cruz Biotechnology (Dallas, TX, USA).
